# Lignin–Cellulose Nanocrystals from Hemp Hurd as Light-Coloured Ultraviolet (UV) Functional Filler for Enhanced Performance of Polyvinyl Alcohol Nanocomposite Films

**DOI:** 10.3390/nano11123425

**Published:** 2021-12-17

**Authors:** Yi Zhang, Abu Naser Md Ahsanul Haque, Maryam Naebe

**Affiliations:** Institute for Frontier Materials, Deakin University, 75 Pigdons Road, Geelong, VIC 3216, Australia; ivf@deakin.edu.au (Y.Z.); a.haque@deakin.edu.au (A.N.M.A.H.)

**Keywords:** light-coloured lignin, cellulose nanocrystals, UV-shielding materials, packaging, sustainability

## Abstract

Lignin is a natural light-coloured ultraviolet (UV) absorber; however, conventional extraction processes usually darken its colour and could be detrimental to its UV-shielding ability. In this study, a sustainable way of fabricating lignin–cellulose nanocrystals (L-CNCs) from hemp hurd is proposed. A homogeneous morphology of the hemp particles was achieved by ball milling, and L-CNCs with high aspect ratio were obtained through mild acid hydrolysis on the ball-milled particles. The L-CNCs were used as filler in polyvinyl alcohol (PVA) film, which produced a light-coloured nanocomposite film with high UV-shielding ability and enhanced tensile properties: the absorption of UV at wavelength of 400 nm and transparency in the visible-light region at wavelength of 550 nm was 116 times and 70% higher than that of pure PVA, respectively. In addition to these advantages, the nanocomposite film showed a water vapour transmission property comparable with commercial food package film, indicating potential applications.

## 1. Introduction

Ultraviolet (UV)-shielding materials are highly beneficial for protecting human skin, food and solar electrical appliances from UV radiation. Though traditional metal oxidant materials (such as TiO_2_ and ZnO) have excellent UV-shielding ability, there are safety concerns [1] over the direct application of these into human-contact products, particularly food packaging films. As such, alternative, bio-based, environmental-friendly materials with UV-shielding properties (such as lignin) have gained significant attention lately [2,3].

Lignin is known for its inherent UV-absorbing capability, with a crosslinked network of different phenolic groups, including syringyl (S) and guaiacyl (G) phenolic groups responsible for UV-shielding properties of lignin [4]. Lignin is abundant and can be extracted from a wide range of natural sources, such as woods and plants. Conventionally, lignin isolation requires treatment chemicals, such as alkalis, acids, enzymes, organic solvents, and ionic liquids [5]. Although these methods are flexible and efficient, they often need harsh chemicals or unique salt (such as ionic liquid), which is expensive. Within these extracting processes, the native phenolic structure of lignin changes, and more chromophore and auxochrome structures are generated [6]. This change in lignin structure is good for the UV-shielding ability, while it will darken the colour of lignin. For example, though native lignin is light brown, alkaline lignin is dark brown [7].

In this paper, lignin is categorised as purified or native lignin [8,9]. The purified lignin is isolated from wood or lignocellulose materials through a complex chemical reaction, and the structure of purified lignin is different depending on its extraction processes. Native lignin is, however, obtained with no (or less) chemical treatments, thus preserving cellulose and hemicellulose inside the lignocellulose materials. Native lignin can be a better choice for light-coloured UV-shielding filler, as less chemical treatment is required. In our previous study [10], we obtained native lignin with the ball milling method. The light-coloured native lignin–cellulose was further added into the PVA matrix as a filler. The film showed high transparency in visible light wavelength (94.3% transmittance at 500 nm) and fully blocked the UV light in wavelength between 200 nm and 231 nm. Though high transparency was achieved by the native lignin–cellulose–PVA composite film, improvement of the UV-shielding and tensile properties is required for future application. In this regard, fabrication of CNCs with native lignin can be effective, since the smaller dimension (nano size) contributes towards both UV-shielding and mechanical strength [11].

Currently, there are only a few reports available on native lignin–cellulose nanocrystals (L-CNCs) preparation, mainly based on acid hydrolysis; however, one of the key drawbacks is the low aspect ratio (around 11) of L-CNCs in the reported methods [12], limiting the application in light-coloured food packaging film. For example, in 2017, Bian et al. [13] first fabricated L-CNCs using maleic acid hydrolysis, achieving an aspect ratio of around 11.25. Later, Agarwal et al. [14] used hydrothermal pre-treatment (has explosion concern) [15] and sulphuric acid hydrolysis, reaching an aspect ratio of around 11.5. Recently, a mixed aspect ratio of L-CNCs ranging from 6.7 to 284 was also reported, related to the mixture of micro- and nano-particles while using an acidic deep eutectic solvent with sulphuric acid [16].

The L-CNCs with high aspect ratio is difficult to achieve due to the crosslink barrier of lignin. The layers of lignin and the crosslinks between cellulose and lignin are two main obstacles of breaking down the cellulose to nanocellulose [17]. Mechanical pre-treatment methods, such as ball milling, have previously been used to break down the lignin network [18] and produce lignin-containing cellulose nanofibers (L-CNFs) which showed a higher aspect ratio [19]. Ball milling is also known as a low cost and environmentally friendly method that retains the chemical structure of native lignin; however, this method has not been used in fabricating L-CNCs, which is a more suitable filler than L-CNFs, because it is likely to be distributed uniformly inside any polymer matrix [20].

Therefore, this study aims to propose a novel method of using a ball milling integrated acid hydrolysis technique to achieve high aspect ratio L-CNCs (preserving the UV-shielding) as a sustainable alternative to the current available techniques. We have chosen hemp hurd, since it is a lignin-rich resource containing around 16–24 wt. % [21,22] of lignin, where 53% of its phenolic groups are S phenolic (highest UV-shielding efficiency among lignin’s phenolic groups), much higher than the S phenolic group in other cellulosic materials [23], e.g., wheat straw, corn stalk, and barely straw. The hemp particle was ball-milled [24] and further hydrolysed under mild acidic conditions at a low temperature (45 °C) to preserve the UV-shielding property and transparency. The influences of acid on the lignin content and UV-shielding performance of L-CNCs were also analysed. Further, to explore potential application, L-CNCs were used as a filler in polyvinyl alcohol (PVA), and the resultant transparent nanocomposite films were characterised for their structural, optical, and tensile properties.

## 2. Materials and Methods

### 2.1. Materials

The hemp hurd (200 g by weight) was dried at 60 °C for 12 h, followed by chopping into a coarse particle with a Fritsch Pulverisette 19 Universal Cutting Mill (sieve diameter of 200 µm). Polyvinyl alcohol solution (PVA) (5 wt. %) was purchased from Flew solution (Queensland, Australia). Sulphuric acid (98 wt. %) was purchased from Chemsupply (Adelaide, Australia).

### 2.2. Fabrication of Native Lignin–Cellulose Nanocrystal (L-CNCs)

Hemp hurd particle (50 g) was wet ball-milled with an Attritor mill (2S, Union Process, USA) at an agitator speed of 280 rpm for 20 h, as reported before [10]. In our previous study with hemp hurd, we have intensively studied the influence of ball-milling time on the crystallinity of hemp [10]. The XRD data of milled hemp hurd particle showed that the crystalline part of hemp hurd reduced from 84.3% to 47% after 20 h milling, while 4 h milling reduced the hemp crystallinity to only 82%. Though some native cellulose was converted into amorphous cellulose, still half remained. The milled hemp particle with the average size of 1.05 μm was kept in the solution to avoid aggregation.

**Figure 1:**
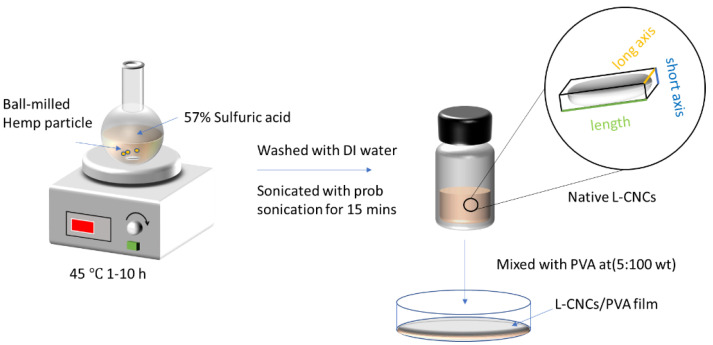
Schematic of fabricating lignin–cellulose nanocrystals at 45 °C and 10 hr hydrolysis time (L-CNCs10) and its subsequent L-CNCs/PVA (5 wt. %) film.

Aqueous solution of 4 g hemp and 25 mL sulphuric acid (64 wt. %) were added into a round bottle flask to hydrolyse for 1, 3, 5, 7, and 10 h under 45 °C, as shown in Figure 1.

The temperature was kept at 45 °C based on the optimised method of fabricating CNCs described in the literature [25]. Though low temperature is preferred to keep lignin intact, a longer hydrolysis time maybe required. According to the different acid hydrolysis treatment times, the samples were named as “L-CNCs1”, “L-CNCs3”, “L-CNCs5”, “L-CNCs7”, and “L-CNCs10” to use hereafter. The concentration of sulphuric acid for all samples was diluted by water from the hemp aqueous solution; thus, it was kept at a rate of 57.25 wt. %. The suspension was diluted with DI water (10 times) to quench the reaction. The L-CNCs solution was centrifuged at 6000 rpm for 5 min on −4 °C three times with DI water to remove additional acid. The residual sample was diluted into 60 mL of DI water and sonicated in a sonication device (Model Q700 Q Sonica) under ultrasonic irradiation (20 kHz, 700 W) for 15 min to well disperse the L-CNCs.

### 2.3. Preparation of L-CNCs/PVA Film

The prepared L-CNCs samples (L-CNCs1, L-CNCs3, L-CNCs5, L-CNCs7, and L-CNCs10) were added to the PVA solution, producing five different mixtures with a weight ratio of 5 wt. %. L-CNCs7, L-CNCs10 were selected to perform further comparison with the addition ratio of 10 wt. % and 20 wt.%. The hemp particle/PVA sample at a ratio of 5 wt.% was prepared from our previous study [10]. This sample was used to compare its properties (UV-shielding ability, tensile property, and water vapour permeability), with those L-CNCs at the same ratio. Therefore, a total of 10 samples were prepared. The mixtures were vibrated on a Vortex Mixer (Ratek, Australia) for dispersion, followed by a separate casting in a Petri dish. The Petri dish was dried in a fume hood overnight at room temperature, and a film with a diameter of 84 cm and 0.040 mm thickness was obtained. Figure 1 shows the preparation process of L-CNCs and L-CNCs/PVA film.

### 2.4. Material Characterisation

#### 2.4.1. Lignin Content

To measure the total lignin in hemp hurd, the extractives were first removed according to NREL/TP-510-42619 [26]. Briefly, the hemp particle, extraction thimble and soxhlet siphon tube were first dried in the oven for a minimum of 12 h. Hemp powder (10 g) and 190 mL 95% ethanol were added in a tared extraction thimble and put into the extraction chamber of the soxhlet extractor. The soxhlet extractor was heated in an oil bath and kept boiling for 10 siphon cycles per hour and refluxed for 24 h. The extracted solid in extraction thimble was transferred into a centrifuge tube and the solid was washed three times. The sample was then dried in the oven at 100 °C to remove free water. Although this method is simple and non-toxic, the amount of lignin in extracted solution is likely to be lower than its true value. This is because some of the lignin (organosolv lignin) [27] is likely to be removed in this stage.

The lignin in hemp hurd included both acid-insoluble lignin and acid soluble lignin. The content of acid-insoluble lignin was determined according to TAPPI T Standard 222 [28]. Dried sample (1 g) was hydrolysed by 15 mL 72% (*w/w*) H_2_SO_4_ for 2 h at 20 °C, followed by adding 575 mL DI water to dilute into 4% (*w/w*) H_2_SO_4_ and boiled for 4 h. The hydrolysis solution and sediment were separated by filtering and washing with hot water. The weight of dried sediment was reported as acid-insoluble lignin. Acid-soluble lignin was measured from the hydrolysis solution by UV–vis spectroscopy, where the absorbance was used to calculate the weight of acid-soluble lignin. The acid-soluble lignin was calculated by the equation from the NREL/TP-510-42618 [29] and was used to understand how much lignin become soluble after longer hydrolysis treatment.

#### 2.4.2. Zeta Potential

The stability and dispersion of L-CNCs in an aqueous solution were evaluated by Zeta potential. The value of Zeta potential was measured on a Zetasizer Nano (ZS, Malvern, UK) at 25 °C. Three measurements were carried out for each sample, and the average was reported. The pH value of L-CNCs suspension was controlled at 7 by diluting the L-CNCs solution 1000 times. This is worth mentioning that the dynamic light scattering (DLS) size is usually used to measure round particles by using the Stokes–Einstein relationship [30]. Since the shape of the particles were not round in this study, we did not use this method for the size measurement.

#### 2.4.3. The Sulphur Content and Yield of L-CNCs

The qualitative analysis of sulphur content in the hemp particle and L-CNCs was analysed by the energy-dispersive X-ray spectroscopy (EDS) method using a scanning electron microscope (SEM) JEOL7800F (Tokyo, Japan). The sulphur content was representing the percentage of the respective sample.

The yield of hemp particles and L-CNCs was measured by using the gravimetric analysis method [31]. The weight of the sample was measured after being dried in the oven for 12 h at 105 °C. The yield of the sample was calculated for the hemp particles.

#### 2.4.4. Atomic Force Microscope (AFM)

To understand the influence of acid hydrolysis on the aspect ratio of L-CNCs, the length, width, and height of L-CNCs were evaluated by AFM (Asylum Research, Santa Barbara, CA, USA). The L-CNCs solution was dropped on the surface of a silicon wafer and dried in a fume hood overnight. The lengths and heights of L-CNCs were measured on 30 random crystals captured by AFM images. The profile drawn by the long axis of the particle represents the length of L-CNCs, and the height represent the maximum value along the long axis [32]. The width of L-CNCs was not accurate due to the artifacts caused by sample-tip convolution [33]. The particle diameter measured directly with AFM may hide undetectable lateral aggregates. Thus, the height measured from AFM was recognised as diameter, and the aspect ratios of L-CNCs were calculated by the length/height (diameter) [34,35]. Data were collected using Nanoscope 8.4 software, and images were analysed using Gwyddion analysis software.

#### 2.4.5. Transmission Electron Microscopy (TEM)

The morphology of L-CNCs was imaged using JEOL TEM LaB6 (Tokyo, Japan). It operated at an acceleration voltage of 200 kV and beam current of 108 μA. The L-CNCs samples were diluted with DI water 1000 times and dropped on a carbon grid. The carbon grid was dried under fume hood for 12 h prior to testing.

#### 2.4.6. Fourier Transform Infrared (FTIR)

A Bruker Vertex spectrometer (70, Billerica, MA, USA) with attenuated total reflectance Fourier-transform infrared (ATR-FTIR) was used to understand the chemical structure of the L-CNCs and L-CNCs/PVA films. A light beam with a wavelength in the range 600 to 4000 cm^−1^ and a resolution of 4 cm^−1^ was used to obtain the spectra. Opus 5.5 software was used to perform the baseline correction of the results. Origin 2020 (OriginLab, MA, USA) was used to normalise and find the peaks. The syringyl-to-guaiacyl ratio was calculated by the intensity of I_1328_/I_1244_ of L-CNCs, where I_1328_ and I_1244_ represented the peak intensity in 1328 cm^−1^ and 1244 cm^−1^, respectively [36].

#### 2.4.7. UV-Shielding Performance of the L-CNCs and the Films

UV-shielding performance of the L-CNCs solution was determined with a UV spectrophotometer (Carry 300 Scan, Varian Inc., Billerica, MA, USA). The spectra of the samples were obtained in a 1 cm quartz cuvette from 200 to 800 nm. To avoid the influence of lignin amount, the data were nominalised per total lignin mass of L-CNCs. Transparency and UV-shielding performance of the films were determined with a UV–VIS–NIR spectrophotometer (Carry 5000 Scan, Varian Inc., Billerica, MA, USA). The spectra of the films were obtained at a scan rate of 600 nm/min from 200 to 800 nm.

#### 2.4.8. X-ray Diffraction (XRD)

The crystallinity of the L-CNCs-PVA film was measured by XRD (Panalytical, Almelo, Netherlands). The intensity in the 2θ range from 7° to 50° were collected in the operating condition at 30 kV and 40 mA. The crystallinity of the film was calculated by Equation (1) [37].

(1) $$Xc=\frac{Ac}{Ac+Aa},$$

where *Xc* is the crystalline fraction, *Ac* represents the crystalline area and *Aa* represents the amorphous area of the film. The crystalline area and amorphous area were deconvoluted through Gaussian function provided in Origin 2020.

#### 2.4.9. Tensile Properties

A universal tensile tester machine (Instron, Norwood, MA, USA) was used to measure Young’s modulus, maximum nominal stress, and elongation at break at 20 ± 2 °C temperature and 62  ±  2% of relative humidity. The film was cut into 30 × 10 mm according to ASTM D882–18 [38]. The gauge length of the sample was 10 mm. The tension rate was kept at 4 mm/min, and the load was recorded with a 100 N load cell. Five specimens of each sample were tested, and the average was reported. Moisture control was done prior to the test, and all the composite films were dried in oven at 30 °C for 30 min before testing.

#### 2.4.10. Water Vapour Permeability

A moisture vapour transmission rate tester (Labthink’s W3/031, Jinan, China) was used to obtain the water vapour permeability (WVP) of L-CNCs/PVA composite films. According to TAPPI T 464 [39], the measurement condition was 90% relative humidity and 38 °C. The water vapour transferred through the films was determined by measuring the weight changes periodically, until a constant weight was reached. Three samples of each film were tested, and the result was obtained from the data average.

#### 2.4.11. The Statistical Analysis

As required, either one-way or two-way ANOVA tests were conducted to understand the difference among samples through IBM SPSS. *p*-value ≤ 0.05 indicates that the difference in data sets was statistically significant, and *p*-value > 0.05 indicates the difference in data sets was not statistically significant.

## 3. Results and Discussion

### 3.1. Lignin Content and Stability of L-CNCs

The acid-soluble and insoluble lignin content (wt. %) of hemp and L-CNCs, along with the zeta potential, are shown in Table 1. The acid-insoluble lignin of hemp was 11.29%, higher than lignocellulose materials such as corn (9.9%), wheat (4.9%), and kale (6.5%) [40] reported in the literature, and its acid-soluble lignin was around 1.4%. The acid-soluble lignin in L-CNCs3, L-CNCs5, and L-CNCs7 was around 2% and increased with longer hydrolysis time, confirming that some of the insoluble lignin was solubilised in acid with longer acid treatment time. Thus, the treatment time was kept lower than 10 h to avoid further transforming lignin into acid soluble lignin.

**Figure 2:**
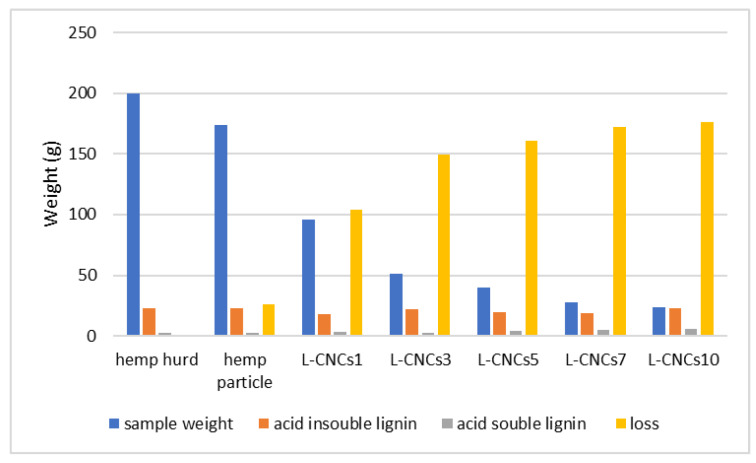
Mass balance calculated from the data reported in Table 1 (all samples have the same total weight).

Though the changes in the amount of acid-insoluble lignin showed fluctuations, overall, it represented an increasing trend. The increase in lignin content was probably related to the loss of cellulose and hemicellulose in the hydrolysis process. The amorphous range of cellulose and hemicellulose were dissolved in the sulphuric acid [41]. This could also be confirmed based on calculated data (Figure 2), as the loss of mass of L-CNCs was more than lignin. Moreover, the molecular weight of sulphated lignin became higher after sulfonation; thus, the amount of acid-insoluble lignin in the samples (measured by weight) increased by the acid treatment compared to untreated hemp particles. On the other hand, the sulfonation group promotes affinity towards water molecules of L-CNCs. The weight of L-CNCs may partly contain water weight, thus leading to weight increase in L-CNCs samples, as shown in Table 1. The rest of the sample would be cellulose nanocrystals, cellulose microparticles, and hemicellulose, depending on the hydrolysis time. The cellulose microparticle and hemicellulose are likely to be decomposed with increasing hydrolysis time [42].

**Figure 3:**
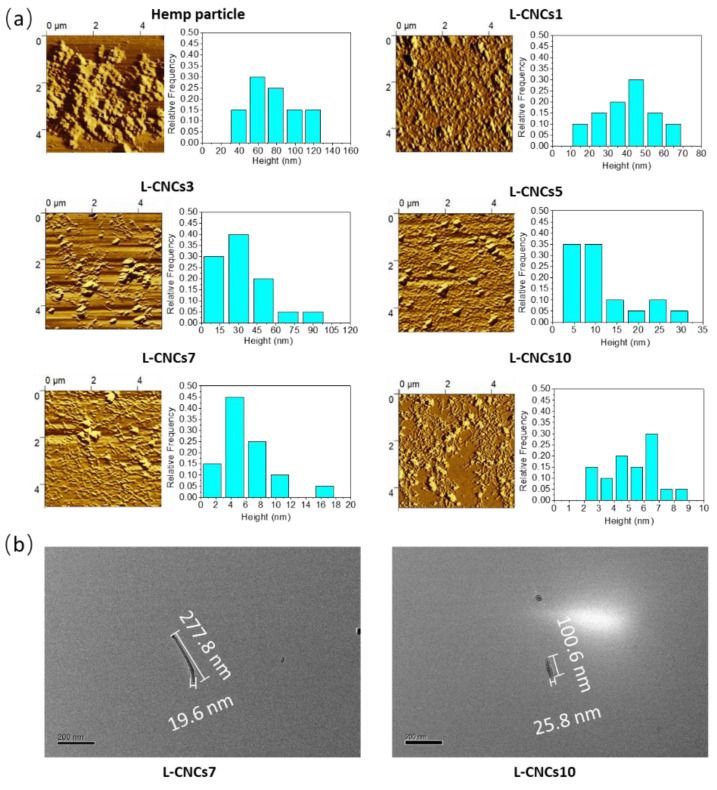
(**a**) AFM image and height of hemp particle and L-CNCs samples. (**b**) TEM image of rod-like L-CNCs7 and L-CNCs10 particle.

The zeta potential was also used to quantitatively characterise the electrostatic stability of L-CNCs. High positive or negative values of zeta potential indicate adequate electrical double layer repulsion, which can avoid possible aggregation. The sulphur content (wt.%) of the samples is presented in Table 1 as a mass percentage. The sulphur content of hemp particles was as low as 0.6 wt. %, while the L-CNCs showed an increasing trend after acid hydrolysis treatment. The L-CNCs10 showed the highest sulphur amount that could be related to the smaller size (will be discussed in morphology and size analysis of L-CNCs section) and longer acid treatment time of L-CNCs10 compared to the rest. The presence of the sulfonation group induces the negative charge of L-CNCs. All L-CNCs aqueous solutions showed negative charges. The zeta potential of L-CNCs1 was −28.63 mV, almost 2.5 times higher than that of hemp particles (−11.36 mV). The zeta potential value of L-CNCs further decreased to −35.43 mV for L-CNCs3, confirming the reduction in the aggregation and uniform dispersions of L-CNCs, as observed in the following AFM images (Figure 3, will be discussed in morphology and size analysis of L-CNCs section). This is because sulphuric acid treatment induces stronger negative surface charges [43] that enhances electrostatic stabilisation of the suspensions.

There was no significant difference in zeta potential values among L-CNCs3, L-CNCs5 and L-CNCs7. The L-CNCs10 showed the highest negative zeta potential indicating the best stability of the L-CNCs samples; however, it lost the highest amount of acid-soluble lignin (3.4%) and showed the highest amount of acid-insoluble lignin (13.2%) among the other L-CNCs. Compared to other L-CNC samples, the hemp particle sample was not treated with acid. The acid was only applied in the measurement process which probably resulted in comparatively lower amount of acid soluble lignin than other samples. Overall, the total lignin amount in the samples was lower than that reported in the literature [21,22]. This difference could be related to the source of lignin and testing method. Moreover, the ethanol extraction pre-treatment could be another reason as some of the lignin was possibly dissolved at that stage [27]. The zeta potential of lignin nanoparticles has been reported between −14 mV and −26 mV in the literature [44]. The measured zeta potential of L-CNCs was almost identical (expect L-CNCs1). This might be related to the extension of surface area, as when particle size reduces the surface area increases. Although the acidity of sample increased, the density of acid did not change.

### 3.2. Morphology and Size Analysis of L-CNCs

The AFM images and height measurements of L-CNCs after subsequent hydrolysis with acid (ball milling assisted acid hydrolysis) are shown in Figure 3a. For comparison, the morphologies of only single L-CNCs7 and L-CNCs10 are shown in Figure 3b. The size and aspect ratio of L-CNCs are summarised in Table 2. The rod-like shape of L-CNCs and some irregular particles are observed for the L-CNCs3, L-CNCs5, L-CNCs7 and L-CNCs10 (Figure 3). The AFM images of L-CNCs are representative of each sample, as a similar morphology was observed after analysing multiple areas. An AFM image of one of the samples (LCNCs7) with a larger area is also shown in Figure S1. The residual lignin could exist in both irregular and rod-like particles. The irregular particle was a partly hydrolysed hemp particle that contained residual lignin. For the rod-like particle, since acid hydrolysis could not dissolve all the lignin, acid-insoluble lignin still existed, which could possibly be attached to the surface of the rod-like particle.

The changes in length (L) and height (H) were found under different hydrolysis times, as shown in Table 2. The hemp particle showed the highest height and decreased through the hydrolysis procedure. With the hydrolysis treatment, irregular particles of hemp generally transformed into rod-like L-CNCs particles, which were obtained in the samples after 3 h hydrolysis. In the first 7 h, the reduction in size of the sample was mainly in the height, with a slow reduction in the length. For the last 3 h (L-CNCs7 and L-CNCs10), the length significantly decreased, but the change in the length was not significantly different in the L-CNCs5 and L-CNCs7. This indicates that the high amount of lignin in hemp has protected cellulose from acid depolymerisation in the first 7 h; lignin acted like glue, adhering cellulose and hemicellulose together in the microfiber [45]. More lignin was soluble in acid with 10 h hydrolysis time, which was also proved by the content of acid-soluble lignin (Table 1).

After depolymerisation of the lignin–carbohydrate complex, cellulose was exposed, resulting in a reduction in length. The length/height aspect ratio (L/H) of L-CNCs7 was the highest (~9 times) among hemp particle and other L-CNCs. The acid hydrolysis treatment from 0 to 7 h resulted in elongated L-CNCs with a higher aspect ratio. With an increased hydrolysis treatment time, the length of L-CNCs further decreased. The higher aspect ratio of L-CNCs would be a benefit for the properties of the composite when reinforced with the polymer. While the aspect ratio of fabricated L-CNCs (using the maleic acid hydrolysis method) has been reported between 9.2 and 11.5 [13], the L/H aspect ratio of the L-CNCs in this study increased from 8.9 to 85.1, which indicated the suitability of this fabrication method in producing L-CNCs with a higher aspect ratio. The higher aspect ratio subsequently resulted in the higher tensile properties and barrier properties of the L-CNCs reinforced composite, which will be discussed later. The enhancement of aspect ratio could be related to the ball milling assistance, since the ball-milled hemp particle showed a uniform morphology. More lignin was exposed after the ball milling, leading to a reduction in height of L-CNCs.

### 3.3. FTIR Analysis

#### 3.3.1. FTIR Analysis of the L-CNCs Particles

**Figure 4:**
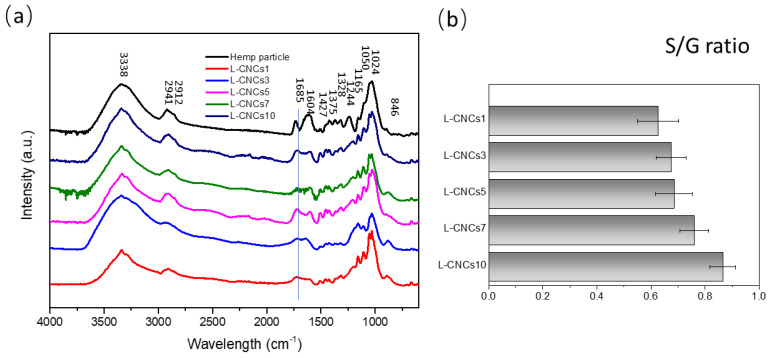
(**a**) FTIR spectrum of hemp particle and L-CNCs. (**b**)The ratio of syringyl to guaiacyl (S/G) in the samples represented by I_1328_/I_1244_.

The chemical change of L-CNCs was analysed by FTIR as shown in Figure 4. The band around 3338 cm^−1^ represent the OH- stretching of intramolecular and intermolecular hydrogen bonds, phenolic groups, phenols from cellulose and lignin [46]. The peaks at 2941 cm^−1^, 2912 cm^−1^, 1244 cm^−1^, and 846 cm^−1^ represented C-H stretching of L-CNCs [18]. Compared with hemp particle, a new peak at 1685 cm^−1^ was observed, and this was assigned to the C=O stretch of alpha, beta-unsaturated ketones [47]. The peak at 1604 cm^−1^ represented the aromatic units [48] in the hemp particle, and it can be observed that it is different from those of L-CNCs. This indicates the structure change in aromatic units of L-CNCs. The peak at 1427 cm^−1^ was related to CH_2_ symmetric bending in lignin [49]. The band at 1373 cm^−1^ represented CH_3_ stretching from cellulose and lignin [50]. The peak at 1350 cm^−1^ and 1050 cm^−1^ represented the S=O stretching [51] of L-CNCs and indicated the present of sulfonate groups. This could be related to the sulfonation of L-CNCs that possibly occurred in the aromatic units of lignin. The bands at 1024 cm^−1^, 1087 cm^−1^, and 1165 cm^−1^ were due to C-O stretching and assigned to the cellulose and hemicellulose. A drop in peak intensity at 1024 cm^−1^ was observed in the L-CNCs3 sample, which indicated the low cellulose content. This sudden drop of cellulose content could be related to the formation of CNCs. As observed in AFM image, the CNCs was started forming after 3 h acid hydrolysis. The peaks at 1328 cm^−1^ and 1244 cm^−1^ were assigned to the syringyl (s) and guaiacyl (G) [52], respectively. As mentioned before, S and G are phenolic groups of lignin, where S phenolic hydroxyl groups contribute more to the UV-shielding properties. The ratio of syringyl to guaiacyl (S/G) was proved to influence the structure of lignin [53]. Lignin is a irregularly branched polyphenolic polyether and its complexity, intricacy and inhomogeneity make the chemical characterisation become challenging [54]. Therefore, the ratio of S/G was used an indicator for UV-shielding properties, usually represented by the I_1328_/I_1244_ [36].

The calculated S/G ratio of hemp particle and L-CNCs are presented in Figure 4. The results indicated an increasing trend of the S/G ratio of L-CNCs with a longer hydrolysis time. The aryl-O-ether linkage (β-O-4) in lignin is sensitive to the acid, which can lead to an increase in phenolic group by cleavage of aryl ether linkages [55] from the G group. The increase in phenolic group can also be observed from the increase intensity of 3338 cm^−1^ peak. Thus, this leads to the enhancement of S/G ratio and UV-shielding properties. The enhanced in S/G ratio was further confirmed by the enhanced UV-shielding properties of L-CNCs, as shown in the following UV–vis result. In addition, the results suggest that the UV-shielding properties of L-CNCs fabricated in this study is higher than softwood (S/G ratio of 0.053 for pine) or grass (S/G ratio 0.433 for switchgrass) and less than hardwood (S/G ratio of 4.25 for eucalyptus) [56].

#### 3.3.2. FTIR Analysis of the L-CNCs/PVA Films

**Figure 5:**
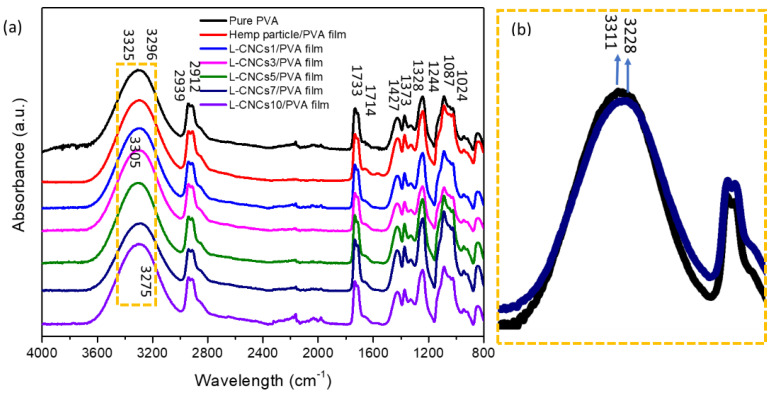
(**a**) FTIR spectrum of pure PVA, hemp particle/PVA and L-CNCs/PVA films. (**b**) Zoom image of pure PVA and L-CNC_S_7/PVA films between 3700 cm^−1^ and 2800 cm^−1^ shown in (**a**).

The hemp particle and L-CNCs were incorporated into PVA as a functional filler. The chemical bonding between particles and PVA were analysed by FTIR, as shown in Figure 5a. The broad peak from 3200–3550 cm^−1^ is due to the intermolecular and intramolecular hydrogen bond [57]. The hydrogen bonding probably took place between the alcoholic (−OH) side groups of PVA and hydroxyl groups CNCs, as they are both hydrophilic [58,59]. PVA showed a broad peak near 3296–3325 cm^−1^, which was identical to the result from Haque et al. [60]. The main peak of hemp particle/PVA was at 3294 cm^−1^. With the hydrolysis procedure, the absorption peak of L-CNCs shifted from 3325 cm^−1^ to 3275 cm^−1^, as shown in Figure 5b. The shifting of the peak may be attributed to the hydrogen bonds formed between hemp particle and PVA, L-CNCs, and PVA. The absorption peak at 1733 cm^−1^ was attributed to the residual acetyl groups in PVA [61]. The rest of the peaks were almost identical to the spectrum of pure PVA. Thus, no other chemical bonding was found between the hemp particle and PVA.

### 3.4. UV-Shielding Analysis

#### 3.4.1. UV-Shielding Performance of Hemp Particle and L-CNCs

**Figure 6:**
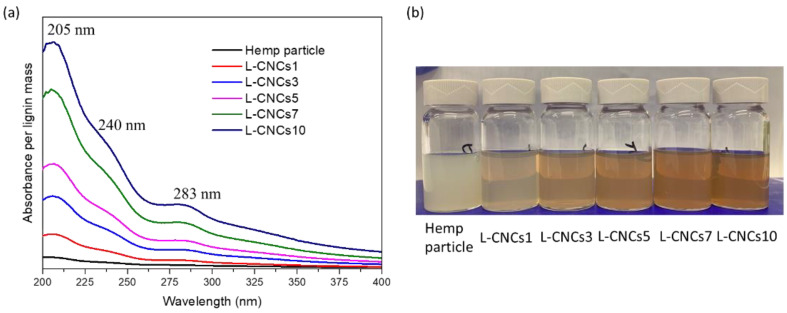
(**a**) UV–vis absorption (normalised per lignin mass in hemp particle and L-CNCs) and (**b**) digital image of hemp particle and L-CNCs.

The UV–vis absorption spectra of hemp particle and L-CNCs are shown in Figure 6a. It was observed that the UV-shielding performance of hemp particle increased through the hydrolysis procedure. The hemp particle and L-CNCs both showed strong peaks at 205 nm and 283 nm, which can be attributed to the aromatic nature of lignin [62]. The absorption peak around 283 nm was assigned to the unconjugated phenolic hydroxyl groups of lignin [57]. With the longer hydrolysis time, sulfonation probably occurred, which was confirmed by the sulphur amount and FTIR spectrum of L-CNCs samples. The UV-shielding ability was related to the chromophores group, such as phenolic units and ketones (as observed from the FTIR result), which has strong UV-shieling ability [63]. Thus, the efficiency of UV-shielding performance increased.

The digital images of hemp particle and L-CNCs are shown in Figure 6b. It can be observed that the colour of L-CNCs became red-brown by the hydrolysis treatment, which is related to the change of the lignin’s chromophore structure. It can also indicate the change of S/G ratio, as the colour of G lignin is yellow-brown, and the colour of S lignin is red-purple [64]. The chromophore groups are the UV-shielding functional group of lignin [4]. The darker colour of L-CNCs also indicates better UV-shielding ability, which is identical to the result from the UV–vis spectra. Longer hydrolysis time could increase the chromophores groups and thus increase the UV-shielding ability of the L-CNCs.

#### 3.4.2. UV-Shielding Performance of the L-CNCs/PVA Films

**Figure 7:**
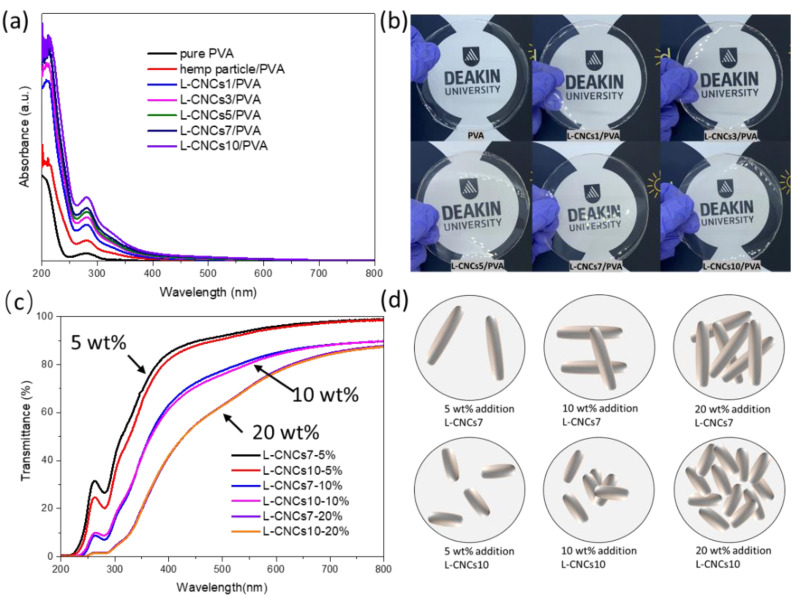
(**a**) Absorption spectra of pure PVA, hemp particle/PVA, and L-CNCs/PVA films (5 wt. %), (**b**) digital images of pure PVA and L-CNCs/PVA films (5 wt. %), (**c**) transmission spectra of L-CNCs7(10)/PVA at 5 wt. %, 10 wt. %, and 20 wt. % ratio and (**d**) Schematic diagram of L-CNCs’ distribution in PVA film.

The hydrolysis influence on the UV-shielding performance was analysed by adding different L-CNCs samples with the same ratio (5 wt. %) in the PVA matrix. The UV-shielding performance and digital image of the prepared films are shown in Figure 7. Figure 7a shows an identical trend in UV absorption of the films to that previously observed for the L-CNCs samples in Figure 6a. While the pure PVA revealed low UV absorption, the UV absorption of composite films was higher due to the presence of hemp particle or L-CNCs. The main absorbance peak of hemp particle/PVA film and L-CNCs/PVA films was around 280 nm related to the lignin. As shown in Figure 7a, the absorption band of PVA at 280 nm was weak, but this peak was mainly intensified by L-CNCs. With the sulphuric acid hydrolysis procedure, the UV-shielding properties of L-CNCs/PVA were stronger than hemp particle/PVA. The better UV-shielding performance could also relate to the size of the hemp particle and L-CNCs. L-CNCs10 showed the smallest particle size (as confirmed by AFM results), and the obtained film resulted in the highest UV absorbance due to the larger surface area and low bulk density of nanoparticles [65]. The better dispersity of L-CNCs in water (discussed in the zeta potential analysis) also contributed to the higher UV-shielding properties. The high zeta potential of L-CNCs resulted in good dispersity in the matrix, as PVA is a water-soluble polymer. The absorbance of all the obtained films showed high visible-light transparency due to the low absorbance in the visible light range (400 nm to 800 nm). Although the colour of L-CNCs was dark brown, as shown in Figure 6b, all the obtained L-CNCs/PVA films were transparent (Figure 7b). This was due to the low amount of L-CNCs added into the PVA matrix, which had little influence on the colour of L-CNCs films. In addition, the nano size of L-CNCs fabricated in this study influenced the lighter colour of the composite. As reported earlier, lignin with nano-size has a lighter colour than the micro size lignin due to the high surface area of lignin nanoparticles, containing more chromophores [66]. As shown in Figure 7b, the L-CNCs/PVA film showed a homogeneous form, and the uniform distribution of L-CNCs in PVA matrix could be related to the hydrophilic nature of both L-CNCs and PVA. Hydrogen bond could be formed between the hydroxyl groups of CNC and alcoholic (−OH) side groups of PVA, as discussed in the FTIR section.

The effect of L-CNCs content on the UV-shielding performance of the composite films is shown in Figure 7c. It was observed that as the amount of L-CNCs increased, the UV-shielding properties of L-CNCs/PVA composite films improved, while the visible-transmittance decreased. The composite films showed identical UV-shielding properties and visible transmittance with L-CNCs7 and L-CNCs10 samples at the ratio of 20 wt. %. Interestingly, at the 10 wt. %, the L-CNCs7/PVA showed more transparency and better UV-shielding properties than the L-CNCs10/PVA film. Although the changes were not significant, they still can be observed from Figure 7c. Transparent UV-shielding lignin composite is very challenging to achieve, as the chromophore groups of lignin is also responsible for the UV-shielding properties. The differences in transparency could be related to the distribution of L-CNCs particles in PVA, as shown in Figure 7d. It has been reported that the aspect ratio can be a beneficial factor for the UV-shielding properties [67]. As discussed in the morphology section, the aspect ratio of L-CNCs7 was observed to be much higher than L-CNCs10. At the ratio of 5 wt. % addition of L-CNCs into PVA film, the L-CNCs thus probably found enough space and distributed evenly in PVA, therefore showing similar UV absorbance results to the L-CNCs solution, as shown in Figure 6a. As schematically shown in Figure 7d, at the ratio of 10 wt. %, the L-CNCs7/PVA film with a higher aspect ratio would have a larger vacant density compared to L-CNCs/PVA film, which results in a lighter colour and better UV-shielding properties. For the addition of 20 wt. % of L-CNCs to PVA, the distribution space was smaller and led to the same UV-shielding performance. For the low L-CNCs content (5 wt. %) PVA composite film, the size of the particles dominated the UV-shielding properties, while with the higher L-CNCs ratio (around 10%) of the film, the aspect ratio became the key factor.

**Figure 8:**
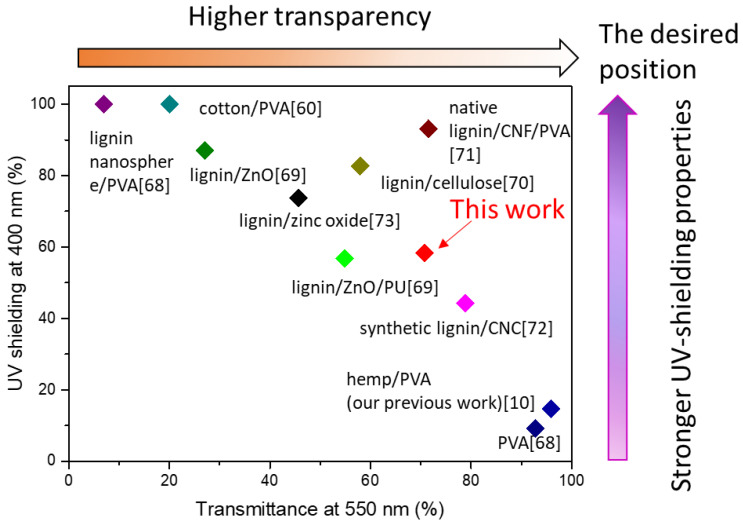
The UV-shielding properties of lignin-CNCs7 20 wt. % film developed in this study in comparison with other lignin UV-shielding composites (this figure was adapted from our previous review paper [66], with the data from reference [10,60,68,69,70,71,72,73]).

UV-shielding properties of some lignin composite materials reported in the literature are shown in Figure 8. For example, the cotton gin waste/PVA film [60] has full UV-shielding at 400 nm, while the transparency at visible light is only around 20%. The L-CNCs7 20 wt. % developed through the current study showed comparable UV-shielding properties with reported materials, while emphasising on the promising potential of native lignin for transparent UV-shielding materials.

### 3.5. Crystallinity Analysis of the L-CNCs/PVA Films

**Figure 9:**
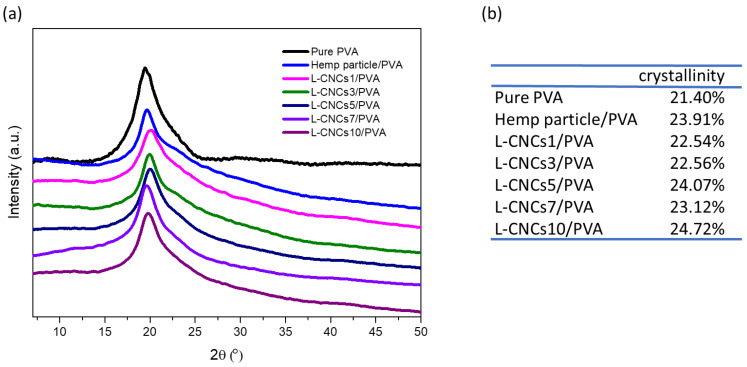
(**a**) XRD spectra and (**b**) crystallinity of pure PVA, hemp particle/PVA and L-CNCs/PVA films (5 wt. %).

Figure 9a shows the XRD pattern of the PVA films (with 5 wt % of L-CNCs). Though we have performed the XRD of L-CNCs, the crystalline part of L-CNCs was not possible to be detected from the spectra. This result suggested that the majority of the cellulose was decomposed after ball milling and long-time acid hydrolysis and resulted in a low yield. Bian et al. [13] also reported that L-CNCs were unable to be detected by XRD spectra due to L-CNCs low yield. The intensities of the crystalline and amorphous part of L-CNCs were very close, which led to a large error after the normalisation. Thus, only the crystallinity analysis of the films is presented here instead. The main peak at 19.3° was assigned to the (101) γ-crystalline phase of the PVA matrix [10]. The main peak of hemp particle is in 22.5°, but it was not observed in the L-CNCs/PVA films, due to the low amount of hemp added to the films, and this is similar to the other reported results from the literature [46,74]. Considering the low yield of L-CNCs, the actual amount of the 5 wt% addition could be 1%. This could also influence the peak of composite. The crystallinity of the films was calculated using Equation (1), where the crystalline and the amorphous areas were obtained through the deconvolution method and are presented in Figure 9b. Addition of either hemp particle or L-CNCs into PVA increased the crystallinity of the films. However, the change in crystallinity did not show that differences between adding hemp particle or L-CNCs, which might be due to the fact that most of the amorphous lignin and hemicellulose of hemp remained in the L-CNCs film. In addition, the amount of hemp particle or L-CNCs added to the PVA matrix was low (5 wt. %), and as mentioned earlier, the main peak of the films was related to the PVA matrix, not hemp.

### 3.6. Tensile Properties of the L-CNCs/PVA Films

**Figure 10:**
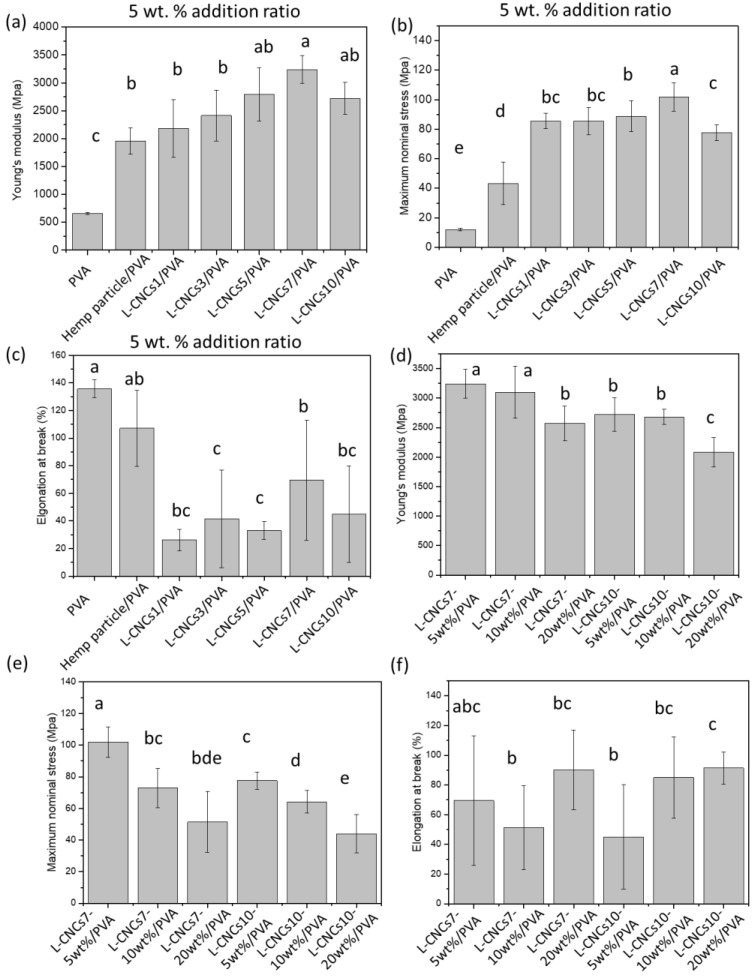
(**a**) Young’s modulus, (**b**) stress, and (**c**) elongation of pure PVA, hemp particle/PVA film and L-CNCs/PVA films at addition ration of 5 wt. %. (**d**) Young’s modulus, (**e**) stress, and (**f**) elongation of L-CNCs7 and L-CNCs10 at different addition ratio (5 wt. %, 10 wt. %, and 20 wt. %). Error bars indicate the standard deviations of the samples. Different superscript letters (a, b, c, d, e) represent statistically significant differences among groups (*p* ≤ 0.05); the superscript letters ab means no significant difference among ab and a or ab and b; the superscript letters bc means no significant difference among bc and b or bc and c; abc means no significant difference among abc and a, abc and b or abc and c; bde means no significant difference among bde and b, bde and d or bde and e.

The influence of L-CNCs on mechanical properties, including Young’s modulus, maximum nominal stress, and elongation at break of PVA film, are shown in Figure 10. Young’s modulus of PVA film was largely influenced by the filler at the ratio of 5 wt.% as shown in Figure 10a. The addition of hemp particle resulted in a significant increase in Young’s modulus of 5 wt.% hemp particle/PVA film by a factor of 4, compared to the pure PVA. While the L-CNCs7/PVA showed the highest Young’s modulus among the films, no significant difference among L-CNCs5/PVA, L-CNCs7/PVA and L-CNCs10/PVA films were observed. A significant difference was found between L-CNCs3 and L-CNCs7. The increase in Young’s modulus in the L-CNCs/PVA films could be the result of two phenomena; (1) the formation of rod-like cellulose nanocrystals as observed by the AFM result and (2) the aspect ratio of hemp particle and L-CNCs [75].

The L-CNCs7 showed a higher aspect ratio of 85.13 compared to those of L-CNCs3 (16.5), as proved by AFM results (Table 2), thus leading to the higher Young’s modulus of the films. This is due to the high aspect ratio of L-CNCs filler, which could possibly form stress concentration zones, and these areas could be transferred with more energy and result in fracture points of the composite. The maximum nominal stress of hemp particle/PVA and L-CNCs/PVA at the ratio of 5 wt. % also showed a significant increase compared to the pure PVA showed in Figure 10b. The maximum nominal stress of L-CNCs7/PVA was the highest and significantly different from other samples. This could be due to the interaction between L-CNCs and PVA. The probable hydrogen bond between L-CNCs7 and PVA could act as transient crosslinks and increase the nominal maximum stress. The elongation of L-CNCs shown in Figure 10c was significantly different from PVA, and only a minor significant difference was found between other L-CNCs samples. This is probably due to the ductility of the film that decreased after adding L-CNCs.

To investigate the influence of filler amount, different contents of L-CNCs7 (highest aspect ratio) and L-CNCs10 (smallest particle size) were also added into PVA with 10 wt. % and 20 wt. %. Young’s modulus, maximum nominal stress, and elongation at break of those PVA composite films are shown in Figure 10d–f. From the two-way ANOVA analysis, Young’s modulus and stress both showed a significant difference between L-CNCs7 and L-CNCs10. Young’s modulus of lignin-CNCs7 20 wt. % films showed no significant difference with lignin-CNCs10 films (5 wt. % and 10 wt. %). The maximum nominal stress decreased with more L-CNCs particles. This was probably due to the increased agglomeration of L-CNCs and a decrease in the degree of dispersion in the PVA composites as shown in Figure S2. For the 20 wt. % film, a filler-to-filler network could be formed due to the high aspect ratio of L-CNCs, while its contribution may be little, and the fracture could probably be occurred due to the particles with irregular shapes. The increased agglomeration of reinforcing agents hindered the effective stress transfer. There was no significant difference found in the elongation at the break of L-CNCs7/PVA films and L-CNCs10/PVA films.

### 3.7. Water Vapour Permeability of the L-CNCs/PVA Films

**Figure 11:**
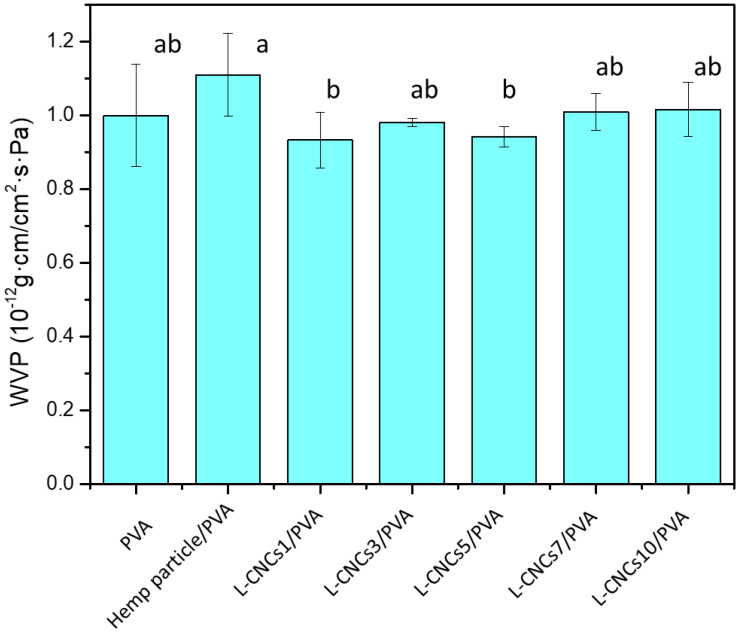
The water vapour permeability of pure PVA, hemp particle/PVA, and 5 wt.% L-CNCs/PVA film. Error bars indicate the standard deviation of samples. Different superscript letters (a, b) represent statistically significant differences among groups (*p* ≤ 0.05); the superscript letters ab means no significant difference between ab and a or ab and b.

Water vapour permeability has been considered an important factor for packaging application, as it can influence the food shelf-life. One of the advantages of PVA is its low water vapour permeability, while adding filler normally influences its permeability. From the literature [76], the barrier property of (ligno)nanocellulose/PVA has improved at a small addition amount. Furthermore, our previous study [10] reported that 5 wt % hemp particle/PVA film showed comparable water vapour permeability to both composite film with 1% and 10% hemp particle addition. Thus, L-CNCs/PVA films at the ratio of 5 wt% (lowest addition amount among our samples) were chosen as representative to study their water vapour permeability. The water vapour permeability of pure PVA, hemp particle/PVA, and L-CNCs/PVA are shown in Figure 11.

Normally, adding fillers could increase the pores of the film, leading to water molecule transfer through the film with less resistance. However, there were no statistical differences found between pure PVA and any of the samples. This could be related to two factors; one is the interaction between L-CNCs and PVA that restricted the passage of water molecules. The water molecules usually move through the amorphous region to permeate from one side to another. The less amorphous region also reduces the diffusion path, which means fewer water molecules pass through. Looking at food packaging materials such as HDPE (high-density polyethene), the water vapour permeability of HDPE has been reported at 1.741–3.482 × 10^−12^ g·cm/(cm^2^·s·Pa) [77], which is comparable to the L-CNCs/PVA films fabricated in this study. This indicates the strong possibility of using L-CNCs/PVA films in food packaging.

## 4. Conclusions

This study demonstrated an innovative fabricating method for high aspect ratio native lignin-cellulose nanocrystals (L-CNCs) by ball milling assisted acid hydrolysis treatment. The proposed fabrication method is more environmentally friendly compared to the current methods, as it was developed using the mild concentration of sulphuric acid (57.25%) at a low temperature (45 °C). Although some acid-soluble lignin was lost through the acid hydrolysis treatment of hemp, the sulfonated L-CNCs showed higher UV absorption than those of hemp particle. Though the colour of L-CNCs was darker than hemp particle, light-coloured UV-shielding nanocomposite film was achieved by the casting of L-CNCs (with different hydrolysis times from 1 to 10 h) with PVA. Among all L-CNCs/PVA films at 5 wt. %, the L-CNCs7/PVA (hydrolysed for 7 h) was highly transparent (94.16% transmission at the wavelength of 550 nm) and showed good UV-shielding properties (73.46% absorption at the wavelength of 280 nm) due to the UV absorption of L-CNCs. At the filler addition of 10 wt. %, the L-CNCs7 (with the highest aspect ratio of 85.13) showed higher transparency and better UV-shielding properties compared to the L-CNCs10, having the smallest size among all L-CNCs. The evaluated tensile properties and water vapour transmission proved the potential application of developed L-CNCs nanocomposite film in food packaging.

## Figures and Tables

**Table 1:** The content of lignin (%), zeta potential and sulphur element of L-CNCs.

Sample Abbreviation	Acid Insoluble Lignin (%)	Acid Soluble Lignin (%)	Zeta Potential Value (mV)	Sulphur Element Content (wt. %)	Yield (%)
Hemp particle	11.3%	1.4%	−11.36 ± 0.64 ^d^	0.6	86.8
L−CNCs1	10.4%	1.8%	−28.63 ± 0.67 ^c^	4.77	48
L−CNCs3	12.7%	1.6%	−35.43 ± 0.76 ^ab^	6.25	25.5
L−CNCs5	11.3%	2.2%	−34.26 ± 1.33 ^b^	7.01	19.8
L−CNCs7	10.6%	2.9%	−34.3 ± 0.88 ^b^	9.74	13.8
L−CNCs10	13.2%	3.4%	−36.43 ± 0.72 ^a^	10.41	11.8

Different superscript letters (a, b, c, d) represent a statistically significant difference among groups (*p*  ≤  0.05); the superscript letters ab mean no significant difference between ab and a or ab and b.

**Table 2:** List of morphological properties of hemp particle and L-CNCs.

Sample Abbreviation	Average Length (L, nm)	Average Height (H, nm)	Aspect Ratios (L/H)
Hemp particle	654.8 ± 339.6 ^a^	73.5 ± 27.3 ^a^	8.9
L-CNCs1	517.3 ± 184.4 ^b^	40.5 ± 14.1 ^b^	12.8
L-CNCs3	498.3 ± 147 ^b^	30.5 ± 20.3 ^c^	16.3
L-CNCs5	486.5 ± 137.4 ^b^	10.5 ± 7.3 ^d^	46.3
L-CNCs7	485.3 ± 172.3 ^b^	5.7 ± 3.3 ^d^	85.1
L-CNCs10	195.8 ± 63.5 ^c^	4.9 ± 1.7 ^d^	40

Different superscript letters (a,b,c,d) represent a statistically significant difference among groups (*p*  <  0.05), the superscript letters cd mean no significant difference between cd and c or cd and d.

## Data Availability

The raw/processed data required to reproduce these findings cannot be shared at this time, as the data also form part of an ongoing study.

## Supplementary Materials

The following are available online at https://www.mdpi.com/article/10.3390/nano11123425/s1, Figure S1: AFM image of L-CNCs7 in the area of 20 × 20 μm^2^, Figure S2: The digital image of L-CNCs/PVA films and the background is white paper with dark words.
