# Supplementary material for: Lignin–Cellulose Nanocrystals from Hemp Hurd as Light-Coloured Ultraviolet (UV) Functional Filler for Enhanced Performance of Polyvinyl Alcohol Nanocomposite Films
Source: Nanomaterials (Basel). 2021 Dec 17;11(12):3425. doi: 10.3390/nano11123425 (PMC8708339; doi:10.3390/nano11123425)
Supplement: Supplementary file 1 [file nanomaterials-11-03425-s001.zip › nanomaterials-1461928-SI.pdf]

nanomaterials

Supplementary Materials

Lignin-Cellulose Nanocrystals from Hemp Hurd as Light-Coloured Ultraviolet (UV) Functional Filler for Enhanced Performance of Polyvinyl Alcohol Nanocomposite Films

Yi Zhang, Abu Naser Md Ahsanul Haque and Maryam Naebe *

Institute for Frontier Materials, Deakin University, 75 Pigdons Road, Geelong, VIC 3216, Australia
* Correspondence: maryam.naebe@deakin.edu.au

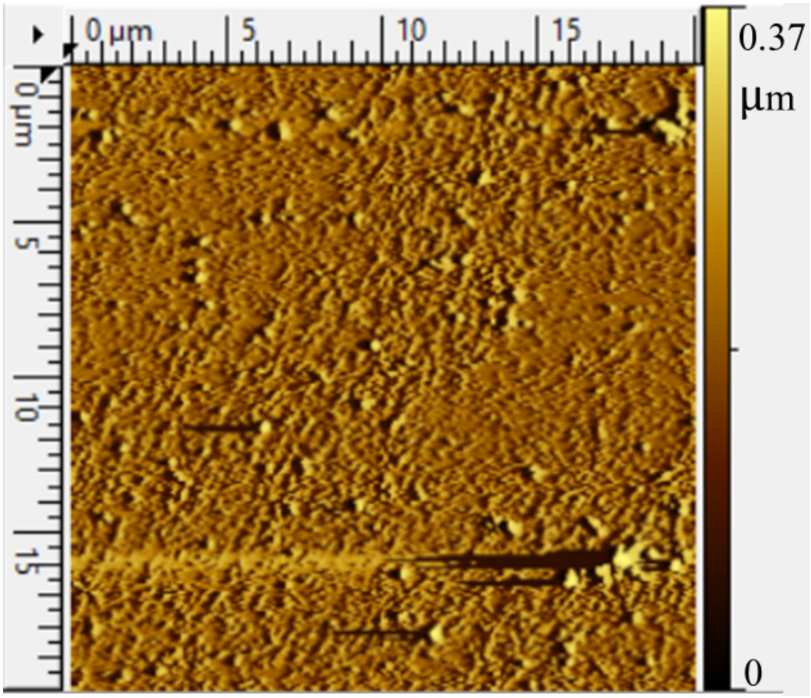

Figure S1. AFM image of L-CNCs7 in the area of 20 × 20 μm^2.

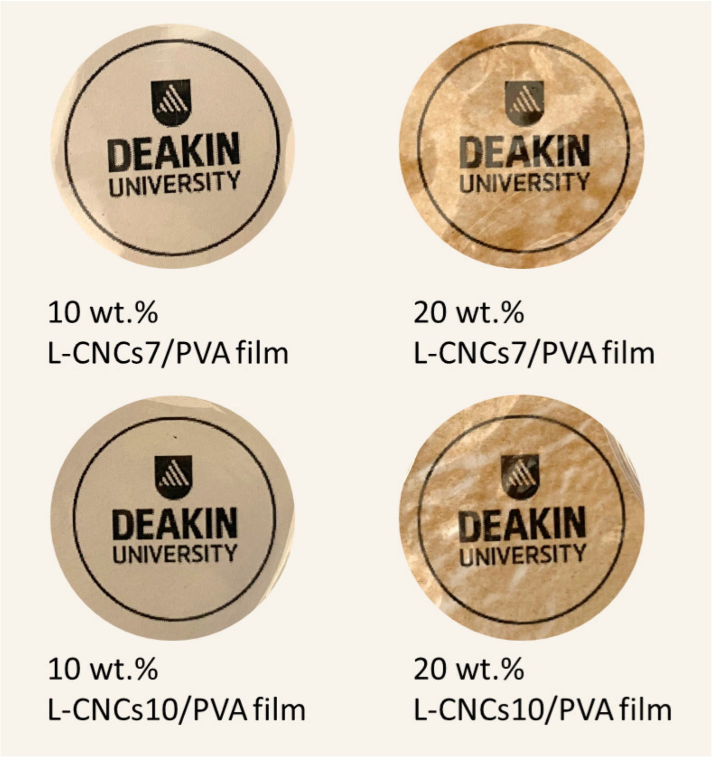

Figure S2. The digital image of L-CNCs/PVA films, the background is white paper with dark words.
